# Patients' preferences in therapeutic decision-making in digestive oncology: a single centre cross-sectional observational study

**DOI:** 10.1038/s41598-023-35407-x

**Published:** 2023-05-26

**Authors:** Pierre Nizet, Charlotte Grivel, Pauline Rabeau, Solange Pecout, Adrien Evin, Sonia Prot Labarthe, Dominique Navas, Fanny Feuillet, Marianne Bourdon, Jean-François Huon

**Affiliations:** 1 Nantes Université, CHU Nantes, Pharmacie, 44000 Nantes, France; 2 UMR INSERM 1246 SPHERE “methodS in Patient-Centered Outcomes and HEalth ResEarch”, Nantes Université, Université de Tours, Nantes, France; 3 Nantes Université, CHU Nantes, Institut Des Maladies De l’Appareil Digestif, 44000 Nantes, France; 4 Nantes Université, CHU Nantes, Service de Soins Palliatifs et de Support, 44000 Nantes, France; 5 Université Paris Cité, INSERM, ECEVE, 75010 Paris, France; 6 Institut de Cancérologie de l’Ouest, Nantes, Angers, France

**Keywords:** Anal cancer, Biliary tract cancer, Colorectal cancer, Pancreatic cancer

## Abstract

Considering the preferences in Shared Decision Making (SDM) of patients with Digestive Cancer (DC) is crucial to ensure the quality of care. To date, there is limited information on preferences in SDM of patients with DC. The objectives of this study were to describe digestive cancer patients’ preference for involvement in therapeutic decision-making and to identify variables associated with these preferences. An observational prospective study in a French university cancer center has been conducted. Patients completed two questionnaires to qualify and quantify their preference for involvement in therapeutic decision-making: the Control Preference Scale (CPS) and the Autonomy Preference Index (API), which is composed of the Decision Making (DM) score and the Information Seeking (IS) score. Associations between these scores and socio-demographic data, disease-related data, coping strategies (Brief-COPE), physical (QLQ-C30) and psychological (HADS) quality of life were tested. One-hundred fifteen patients returned the questionnaires. The majority of patients reported a passive (49.1%) or a collaborative (43.0%) CPS status. The mean DM score was 39.4 Variables associated with decision-making preferences were occupational status and time since diagnosis. The identification of variables associated with patients' preferences for involvement in decision-making can help make clinicians aware of patients' needs and wishes. However, it can only be determined by interviewing the patient individually.

## Introduction

Digestive Cancers (DC) are one of the most frequently diagnosed and deadly human cancers worldwide, notably because they can occur at any anatomic sites of the digestive system^1,2^. In digestive oncology, the diversity of eligible and relevant treatments for a patient’s tumor can lead the patient to express her/his preferences^3^. For instance, patients with colorectal cancer may express preferences for receiving or not receiving adjuvant chemotherapy, while patients with pancreatic cancer may express preferences for a protocol over another based on their intensity. Patients suffering from digestive cancers can face major difficulties due to the side effects of treatments^4^. They may be more inclined to prioritize longevity or quality of life based on sociodemographic and health factors^5^. Therefore, patients’ preferences are crucial in Shared Decision Making (SDM), which is integrated into the decision-making process for cancer treatment^6^. Yet, to date, there is limited information on preferences in SDM of patients with digestive cancers. SDM has received increased attention in recent decades as a fundamental component of patient-centered care, moving away from the paternalism long practiced in care^7^.It is based an active and collaborative relationship between a health care professional and a patient. In France, SDM has officially become part of the relationship between the healthcare provider and the patient since the law of March 4th 2002^8,9^ relating to garding the rights of patients and the quality of the health care system. SDM promotes patient autonomy, which is one of the four principles of biomedical ethics^10^. To date, the principles and core elements of SDM have been summarized and are well documented. In 2006, a systematic review of the literature exploring the conceptual definitions of SDM resulted in an integrative theoretical model suggesting nine essential steps from the physician alone to patient alone via equal sharing^11^. In clinical practice, Elwyn et al*.* proposed a 3-step model: (1) introducing choice, (2) describing options, (3) helping patients explore preferences and make decisions^12^. More recently, authors described four major steps of SDM which seem necessary for it realization: (1) make patients aware that they have a choice, (2) examine the different alternatives and discuss the consequences of each including their probabilities, (3) take into account and support patient’s values, (4) make a decision based on informed preferences^13^.

Previous research has shown that SDM was associated with positive outcomes. SDM may favor improved quality of life (QoL) of patients with chronic disease, including cancer, and their relatives^14–16^, could promote adherence to prescribed medication, improve patient satisfaction, and reduce physician or emergency department visits^17,18^. In parallel, research has found that the patient's involvement in decision making can create a sense of self-blame and regret for them if a treatment fails^19,20^.Thus the SDM process is particularly complex in oncology. The physician and the patient must simultaneously consider advances in treatment, toxicity and uncertainty about the effectiveness of treatment^14,21,22^ to make a decision. diagnostic and therapeutic decisions in oncology must therefore be discussed beforehand at a Multidisciplinary Consultation Meeting (MCM)^23^ during which patients are usually not involved^24^. A MCM in the initial management of a patient with cancer ensures the quality of care since it aims at determining the best therapeutic strategy; ensuring the duty of competence towards the patient, and guaranteeing, the quality of the decision independently of the opinion of the referring oncologist. The decision-making process involves a rational distancing^25^ that takes little account of patients’ preferences at first glance. Nevertheless, physician and patient can share information and interact before or after a MCM^26^ and a recent study has shown that patient participation in MCM could not change their desire for information^27^.

Therefore, SDM is complex and has been little studied in the field of digestive oncology, while considering the preferences in SDM of patients with digestive cancer is crucial to ensure the quality of care. This paper aims to describe DC patients’ preference for involvement in therapeutic decision-making to highlight their preferences. Associations between these preferences and socio-demographic, socio-economic, QoL or coping strategy variables are also examined to identify variables that may guide clinicians toward individualized discussions with patients. A recent systematic review has highlighted that cancer patients did not experience a shared decisional for treatment while they would have preferred^28^. Our study will give new insights into the preferences of DC patients with the aim of encouraging their implementation in clinical practice.

## Methods

An observational prospective study in a French university cancer center with in and out patients has been conducted. Patients were eligible for the study if they were over 18 years old and received a treatment for digestive cancer. Exclusion criteria were restricted to difficulties in completing the questionnaires (e.g. dementia, French language barriers, reading difficulty) to avoid response bias related to hetero-administration. Eligible patients read an information letter introducing the study and its objectives. If they agreed to participate, they were included into the study and completed the questionnaires. The patients were included regardless of time since diagnosis and prognosis. The study was approved by the local ethic group in August 2021.

### Data collection

Using a self-administered questionnaire, DC patients were asked to provide socio-demographic information including marital and occupational status. Additional information regarding socio-demographic status (age, sex) and medical information (cancer type and prognosis, date of cancer diagnosis, date of first treatment, inclusion in a clinical trial) were collected from the medical charts. The prognosis was measured by the metastatic status of the cancer. On-going medical treatments and comorbidities were collected from the patient's medical history.

Then DC patients completed five self-report questionnaires. The five questionnaires used were chosen because they are frequently used in the literature and they were validated in their French version beforehand.

Preferences for involvement in medical decision-making was assessed using the *Control Preference Scale* (CPS)^29,30^. Patients were asked to select from five statements that best reflected their preference for involvement in decision-making. These statements describe a preference for an active, a collaborative, or a passive role in decision-making about treatment strategy.

Expectations regarding information and preference for decision-sharing were assessed using the *Autonomy Preference Index* (API)^31,32^. Responses were reported on a 5-point Likert scale (a score of 5 indicating the highest preference). The computation of the API scores was explained in the original publication as follows: Information-Seeking (IS) and Decision Making (DM) scores are calculated as the sums of the 8 and 6 responses, respectively, adjusted linearly ranging from 0 to 100 (the strongest possible desire). The IS score quantifies the desire for information, while the DM score quantifies the preference for involvement in therapeutic decision-making.

Depression and anxiety were assessed using the *Hospital Anxiety Depression Scale* (HADS)^33,34^, each dimension comprising 7 questions with 4 response modalities. It is possible to calculate a depression score and an anxiety score, each between 0 and 21. For each dimension, a score less than or equal to 7 indicates *no symptomatology*, a score between 8 and 10 reflects *doubtful symptomatology* and a score greater than or equal to 11 indicates *definite symptomatology*.

Health-related QoL was evaluated using the *European Organization for Research and Treatment of Cancer Quality of Life Questionnaire* (EORTC QLQ-C30)^35^. It consists of 30 items measuring global health; physical, role, emotional, cognitive and social functioning; symptoms (nausea, vomiting, fatigue, pain, dyspnea, sleep, appetite, transit) and a financial dimension. Symptoms and the financial dimension were not explored in this study. Scores range from 0 to 100. Higher scores represent higher levels of global health and functioning.

Coping strategies were assessed using the *Brief*-*COPE* (*Coping Orientation to Problems Experienced*)^36,37^. Main instructions of the Brief-COPE were situational, i.e. in relation with the context of the cancer diagnosis. This questionnaire evaluates fourteen adjustment strategies to cope with stress, using 28 items. DC patients had to answer on a 4-point Likert scale for each item. The higher the score for a strategy, the more the patient used it to cope with cancer. In this study, we grouped together several strategies according to the model of *Baumstarck *et al*.*^38^. We obtained four strategy groups, namely* “*Seeking social support*”* (i.e. venting, emotional support, instrumental support, religion)*; “*Problem solving*”* (i.e. active coping, planning)*; “*Avoidance*”* (behavioral disengagement, distraction, substance use, denial, blame) *and “*Positive thinking*”* (humor, positive reinterpretation, acceptance).

### Statistical analysis

Categorical variables (i.e. CPS status, gender, occupational status, marital status, treated comorbidities, type of disease, metastatic status, inclusion in a clinical trial, depression, anxiety) were presented in numbers and percentages. Continuous variables (age, time since diagnosis, time since first treatment, API IS scores, QoL functioning, coping strategies*)* were introduced by means and standard deviations or medians and quartiles. In order to identify variables related to the desire for involvement in therapeutic decision-making, a univariate analysis was performed between the different variables and the CPS status stated by the patient. Categorical variables were compared by Chi^2^ or Fisher tests. Continuous variables were compared by non-parametric Kruskal–Wallis tests. A univariate analysis was also performed between the different variables and the API DM score. Categorical variables were compared by non-parametric Wilcoxon or Kruskal–Wallis tests. Continuous variables were compared using Pearson correlation coefficients.

### Ethics approval

Approval was obtained from the ethics committee « Groupe Nantais d’Ethique dans le Domaine de la Santé » (GNEDS). The procedures used in this study adhere to the tenets of the Declaration of Helsinki.

### Consent to participate

Informed consent was obtained from all individual participants included in the study.

## Results

From January to March 2022, 135 DC patients were included. Of these, 115 (85.2%) returned questionnaires.

The sociodemographic and disease-related characteristics of DC patients are introduced in Table 1. No data were missing. The average age of DC patients in the population was 64.7 years (σ = 10.9). Seventy-six (66.1%) were male. Seventy-six (66.1%) patients were retired, 12 (10.4%) were unemployed and 27 (23.5%) were employed. More than 80% of the patients had at least one comorbidity that required chronic treatment in addition to cancer treatment. Fifty-two (45.2%) patients had colorectal cancer, 29 (25.2%) had hepatocellular carcinoma and 22 (19.1%) had pancreatic cancer. A majority of patients had metastatic cancer (63.5%) and a minority were involved in a clinical trial (17.4%). The average time since diagnosis was 20.7 months (σ = 21.5) and the average time since the first treatment at study completion was 4.2 months (σ = 5.2).

**Table 1:** General characteristics of patients.

		N = 115
Average age in years (σ)		64.7 (10.9)
Gender	Women	39 (33.9%)
	Men	76 (66.1%)
Married	Yes	86 (74.8%)
	No	29 (25.2%)
Occupational status	Employed	27 (23.5%)
	Unemployed	12 (10.4%)
	Retired	76 (66.1%)
Comorbidities treated with medication	Yes	93 (80.9%)
	No	22 (19.1%)
Type of cancer	Hepatocellular carcinoma	29 (25.2%)
	Cholangiocarcinoma	8 (7.0%)
	Colorectal cancer	52 (45.2%)
	Stomach cancer	2 (1.7%)
	Pancreatic cancer	22 (19.1%)
	Oesophageal cancer	2 (1.7%)
Metastatic stage	Yes	73 (63.5%)
	No	42 (36.5%)
Clinical trial	Yes	20 (17.4%)
	No	95 (82.6%)
Average time, in months, since diagnosis (σ)		20.7 (21.5)
Average time, in months, since first treatment (σ)		4.2 (5.2)

Regarding the CPS, nine patients (7.9%) declared themselves to be *active:* one patient answered "*I prefer to make the decision about the treatment I will receive*", the others answered "*I prefer to make the final decision about my treatment after serious consideration of my doctor's opinion*" which indicates a pre-notion of patient-physician collaboration in decision-making. Forty-nine patients (43.0%) declared themselves to be collaborative, i.e. they preferred to share the decision of treatment with their physician. Finally, 56 patients (49.1%) were passive: half preferred the physician to make the final decision on which treatment was used, considering their opinion, and the other half of the patients preferred the physician to make all decisions.

The mean API Decision Making (API DM) score was 39.4 (σ = 17.4).

### Univariate analysis

The results are presented in Tables 2 and 3. Occupational status was significantly related to CPS status (p < 0.05). The proportion of retired patients was higher in the group of patients who reported a passive CPS status (71.4%), compared to those who reported an active (55.6%) or a collaborative (63.3%) CPS status. The proportion of employed patients was higher in the group of patients reporting an active CPS status (33.3%), compared to those reporting a collaborative (18.4%) or a passive (26.8%) CPS status. The variable "time since diagnosis" (*p* < 0.05) was also significantly related to CPS status. The median time since diagnosis was higher in the group of patients who reported a collaborative CPS status (23.7 months), compared to those who reported an active (7.1 months) or a passive (10.7 months) CPS status. None of the other tested variables was significantly related to self-reported CPS status and none were related to the API DM score.

**Table 2:** Univariate analysis of categorical variables according to the CPS status and the median API DM score (Chi2 test or Fisher's exact test) among the 115 patients.

		CPS				API	
		Active (n = 9)	Collaborative (n = 49)	Passive (n = 56)	*p*	Median DM score [Q1;Q3]	*p*
Gender	Women	2 (22.2%)	18 (36.7%)	19 (33.9%)	0.76	41.7 [33.3;58.3]	0.09
	Men	7 (77.8%)	31 (63.3%)	37 (66.1%)		37.5 [25.0;45.8]	
Married	Yes	6 (66.7%)	35 (71.4%)	45 (80.4%)	0.47	41.7 [29.2;50.0]	0.77
	No	3 (33.3%)	14 (28.6%)	11 (19.6%)		37.5 [29.2;45.8]	
Occupational status	Employed	3 (33.3%)	9 (18.4%)	15 (26.8%)	**0.0325**	41.7 [29.2;58.3]	0.66
	Unemployed	1 (11.1%)	9 (18.4%)	1 (1.8%)		39.6 [27.1;52.1]	
	Retired	5 (55.6%)	31 (63.3%)	40 (71.4%)		37.5 [27.1;45.8]	
Comorbidities treated by medication	Yes	5 (55.6%)	41 (83.7%)	47 (83.9%)	0.14	41.7 [37.5;54.2]	0.18
	No	4 (44.4%)	8 (16.3%)	9 (16.1%)		37.5 [25.0;45.8]	
Type of cancer	Hepatocellular carcinoma	3 (33.3%)	11 (22.4%)	15 (26.8%)	0.91	37.5 [25.0;45.8]	0.44
	Cholangiocarcinoma	0 (0.00%)	3 (6.1%)	5 (8.9%)		39.6 [25.0;45.8]	
	Colorectal cancer	5 (55.6%)	25 (51.0%)	21 (37.5%)		41.7 [31.3;52.1]	
	Stomach cancer	0 (0.00%)	0 (0.00%)	2 (3.6%)		29.2 [16.7;41.7]	
	Pancreatic cancer	1 (11.1%)	9 (18.4%)	12 (21.4%)		37.5 [25.0;54.2]	
	Oesophageal cancer	0 (0.00%)	1 (2.0%)	1 (1.8%)		22.9 [20.8;25.0]	
Metastatic status	Yes	5 (55.6%)	34 (69.4%)	33 (58.9%)	0.49	37.5 [29.2;50.0]	0.38
	No	4 (44.4%)	15 (30.6%)	23 (41.1%)		37.5 [25.0;45.8]	
Clinical trial	Yes	1 (11.1%)	11 (22.4%)	8 (14.3%)	0.56	37.5 [25.0;45.8]	0.19
	No	8 (88.9%)	38 (77.6%)	48 (85.7%)		41.7 [35.4;56.3]	
HADS anxiety	No symptom (≤ 7)	6 (66.7%)	23 (46.9%)	31 (55.4%)	0.63	37.5 [27.1;45.8]	0.93
	Doubtful symptomatology (8–10)	1 (11.1%)	17 (34.7%)	17 (30.4%)		39.6 [29.2;50.0]	
	Definite symptomatology (≥ 11)	2 (22.2%)	9 (18.4%)	8 (14.3%)		37.5 [25.0;54.2]	
HADS depression	No symptom (≤ 7)	7 (77.8%)	35 (71.4%)	41 (73.2%)	0.98	37.5 [25.0;50.0]	0.76
	Doubtful symptomatology (8–10)	1 (11.1%)	9 (18.4%)	8 (14.3%)		37.5 [29.2;41.7]	
	Definite symptomatology (≥ 11)	1 (11.1%)	5 (10.2%)	7 (12.5%)		37.5 [33.3;50.0]	

*CPS* control preference scale, *API* autonomy preference index, *DM* decision making, *HADS* hospital anxiety and depression scale.

Significant values are given in bold.

**Table 3:** Univariate analysis of continuous variables according to the CPS status (Kruskal Wallis test or non-parametric Wilcoxon tests) and the API DM score (Pearson correlation coefficients) among the 115 patients.

Median [Q1; Q3]	CPS				API	
	Active (n = 9)	Collaborative (n = 49)	Passive (n = 56)	*p*	Pearson correlation coefficient DM score	*p*
Age (years)	63.0 [62.2; 68.1]	66.7 [58.2; 70.2]	69.1 [58.5; 73.8]	0.67	− 0.09	0.31
Time since diagnosis (months)	7.1 [2.5; 19.1]	23.7 [8.7; 33.4]	10.7 [5.0; 19.1]	**0.0185**	0.02	0.82
Time since first treatment (months)	3.2 [0.0; 3.7]	2.3 [0.5; 8.0]	2.3 [0.7; 5.1]	0.54	0.12	0.19
API Information-seeking (IS)	85.0 [75.0; 92.5]	87.5 [80.0; 95.0]	90.0 [82.5; 97.5]	0.44	− 0.10	0.30
QLQC30 Global health status	66.7 [33.3; 83.3]	66.7 [50.0; 75.0]	66.7 [50.0; 83.3]	0.44	− 0.003	0.97
QLQC30 Physical functioning	80.0 [53.3; 80.0]	86.7 [66.7; 93.3]	80.0 [66.7; 93.3]	0.31	− 0.03	0.76
QLQC30 Role functioning	66.7 [0.0; 66.7]	66.7 [50.0; 100.0]	66.7 [33.3; 100.0]	0.26	− 0.13	0.17
QLQC30 Emotional functioning	83.3 [83.3; 91.7]	75.0 [66.7; 91.7]	83.3 [58.3; 91.7]	0.52	0.01	0.95
QLQC30 Cognitive functioning	83.3 [33.3; 100.0]	83.3 [66.7; 100.0]	83.3 [66.7; 100.0]	0.73	− 0.02	0.84
QLQC30 Social functioning	50.0 [33.3; 66.7]	66.7 [50.0; 100.0]	66.7 [50.0; 100.0]	0.30	− 0.14	0.13
Brief-COPE—Seeking social support	46.9 [40.6; 50.0]	43.8 [37.5; 62.5]	43.8 [35.9; 60.9]	0.56	− 0.001	0.99
Brief-COPE—Problem solving	43.8 [31.3; 50.0]	50.0 [43.8; 62.5]	56.3 [43.8; 75.0]	0.08	− 0.11	0.23
Brief-COPE—Avoidance	37.5 [32.5; 42.5]	37.5 [32.5; 47.5]	40.0 [32.5; 46.3]	0.95	0.03	0.72
Brief-COPE—Positive thinking	58.3 [45.8; 62.5]	62.5 [54.2; 75.0]	58.3 [47.9; 68.8]	0.18	− 0.02	0.83

*CPS* control preference scale, *API* autonomy preference index, *DM* decision making, *QLQC30* quality of life questionnaire, *Brief-COPE* brief coping orientation to problems experienced.

Significant values are given in bold.

## Discussion

The objectives of this study were to describe DC patients' preferences for involvement in therapeutic decision-making and to identify variables associated with these preferences.

### Control preference scale

In this study, the proportion of patients who preferred a passive or a collaborative role in therapeutic decision-making were similar (49.1% and 43.0%, respectively). The proportion of patients who preferred an active role was low (7.9%). The distribution of roles differs greatly from one study to another. For instance, Elkin et al. conducted a study in metastatic colorectal cancer patients over 70 years old and found that 52% of patients preferred a passive role, 23% of patients a collaborative role and 25% of patients an active role^3^. In contrast, a study conducted by Bruera et al. in patients suffering from mixed types of cancer reported a high preference for the collaborative role (63%)^39^. A meta-analysis of cancer patients' preferred role in treatment decision-making indicates that half of patients preferred a collaborative role^40^. These discrepancies could be related to the characteristics of the population studies, which widely differ. For instance, the average age of the Elkin et al*.* population was higher than the mean age of the population, which might explain the lower preference for the collaborative role. A systematic review of preferences for involvement in therapeutic decision making in cancer patients showed heterogeneous results across studies^41^. Furthermore, it has been found in several studies that women are generally collaborative^42,43^, confirmed in the female dominated population of Bruera et al. Noteworthy, differences in care systems according to countries that allow more or less room for patient preferences could also be associated with these discrepancies. In addition, Truglio-Londrigan et al*.* have found differences in patient involvement preferences according to the different cultures^44^*.* Turning now to the variables significantly associated to the preferences in our study, occupational status was related to the preference of involvement. The proportion of patients reporting a passive role in decision-making was higher among retired DC patients than among employed DC patients. Perhaps less involvement in occupational life and less responsibility would favor increased passivity in decision-making. The variable "time since diagnosis" was also related to CPS status. Patients who reported a preference for collaborative decision-making had their diagnosis for a longer time. Patients' preferences are likely to develop over time as they gain experience and may change at different stages^42^. Increased awareness about patients’ preferences for involvement could better support the expression of patient preferences and their identification by the oncologist could support shared decision-making.

### Autonomy preference index DM score

The mean DM score in our study was 39.4. Another study in primary care found scores between 36.6 and 50.6 depending on the motives for consultation. Lower scores were associated with increased severity of the disease and older age^45^. Considering digestive cancers as severe diseases, the average DM score in our study could be interpreted consistent with this study. None of the variables tested in this study were significantly associated with the DM score.

The desire for information characterized by the API IS score was neither correlated with the API DM score nor with the CPS status. A key step in SDM is the two-way transfer of information between the patient and the healthcare professional^46,47^. Therefore, we expected that the more patients would like to be involved in decision-making, the more information they would like to have about the treatment and the disease, as described in previous studies^48–50^. A systematic review identified several factors associated with the need for health information, such as gender, age, education level, time since diagnosis, course of the disease and psychological QoL, but no associations were found with the desire for involvement in decision-making^51^.

Anxiety, depression and health-related QoL were not significantly correlated with the preference for involvement in decision-making in our study. We hypothesized that non-anxious and non-depressed patients would like to be more involved in decision-making, although few studies have found a link between the desire for involvement in therapeutic decision making and psychological disorders^41^. Anxiety is characterized by ruminations that can interfere with decision-making^52^ and depression is characterized by a state of sadness that leads to withdrawal and low commitment in actions^53^.

The originality of this descriptive study was to identify the DC patients' preferences in relation with their involvement in therapeutic decision-making. Our preliminary study can help to determine variables that have to be taken into account in further in-depth investigations relevant for clinical practice. Previous studies showed that patients often do not have the decision-making roles they would like to have^54,55^ or that physicians' perceptions of patients' preferences can be wrong^3^. In parallel, other studies have shown that the more a decision is collaborative, the more a patient is satisfied with the choice of treatment and the decision-making process^56,57^. In the current context of changing medical culture, shared decision-making is often considered as the ideal model for the physician–patient relationship. Medical culture is moving from medical paternalism^58^, which means that physicians make decisions with little room for patient preferences, to a system that includes patient autonomy and informed participation in medical decisions^59^. Shared decision-making honors the knowledge and the skills of the physician on one side and the rights of patients to have their preferences informed by decisions on the other side. However, shared decision-making is not wanted by all patients, especially those who prefer to have a passive role. Moreover, the experience of a specific disease is likely to affect patients' preferences and their desire for involved decision-making. This desire can evolve over time and change with the stage of disease^42^.

In France, the 2002 law strengthens the patients’ right to make decisions and gives them legislative tools to promote their autonomy. This autonomy requires an understanding of the situation and of the therapeutic possibilities in order to make a choice. In this context, the law stresses the need to inform and on the quality of the delivered information. The oncologist must explain to the patient the situation and his/her reasoning from the assessment, *i.e.* treatment options, benefit/risk balance of each option with the therapeutic aim. This information is necessary to promote patient involvement in decision-making. It has to be accurate and individually adapted to the patient to facilitate cognitive and emotional processing. The opinion of an oncologist can differ from the opinion of a patient if a common goal was not previously defined. This step is particularly significant as they potentially rely on different values.

Identifying variables associated with patients' preferences for participation in decision-making can help make clinicians aware of their patients' preferences. Involving patients in decision-making is one of the many ways to improve quality of care^60^. However, there is also a risk that it leads to predicting preferences or profiling patients based on assumptions. The most important finding of this study is that patients' preference for involvement in treatment decision-making is highly variable and cannot be predicted from the study data alone. It can only be determined by interviewing the patient individually. Especially as these preferences may vary over time, we recommend that clinicians regularly assess patients' preferences for involvement to meet their expectations.

### Limitations and perspectives

There is a possible inclusion bias since patients who are interested in shared decision-making might have been over-represented. A second limitation was the heterogeneity of time between the diagnosis, the last therapeutic decision and the completion of questionnaires. We chose to include patients regardless of the time elapsed since diagnosis or since the last decision to measure the relationship between these variables and the preference for involvement in decision making. Given that he experience of the disease may affect patients' preferences for involvement in decision making^42^, it would be beneficial to include patients at time of cancer diagnosis and to follow them over their course of treatment and recovery. This may enable an increased insight into factors associated with preferences and to study change in preferences over time. Thirdly, recall bias may have affected the response to the patient questionnaires^61^. Patients with chronic diseases tend to overestimate their past QoL^62,63^.Finally, in the literature, level of education and health literacy were positively correlated with the preference for an active or a collaborative role in decision-making^43,64,65^. It could be interesting to conduct an ancillary study using semi-structured interviews to explore the experience of patients regarding their desire for involvement. In addition, complementary qualitative data could help to explain quantitative results and to identify relevant variables to investigate in future studies.

## Conclusion

These findings can guide clinicians toward individualized discussions with their patients and bring them more insights into the patient's preferences for involvement in decision making throughout the course of care, particularly when a therapeutic decision is about to be made. Identifying patients' preferences in an individualized manner will allow physicians to respect the patient's desire for involvement in decision-making.

## Data Availability

The datasets used and analyzed during the current study available from the corresponding author on reasonable request.
